# Absolute binding free energy calculations improve enrichment of actives in virtual compound screening

**DOI:** 10.1038/s41598-022-17480-w

**Published:** 2022-08-10

**Authors:** Mudong Feng, Germano Heinzelmann, Michael K. Gilson

**Affiliations:** 1 Department of Chemistry and Biochemistry, and Skaggs School of Pharmacy and Pharmaceutical Sciences, UC San Diego, La Jolla, CA 92093 USA; 2 Departamento de Física, Universidade Federal de Santa Catarina, Florianópolis, Santa Catarina Brazil

**Keywords:** Virtual screening, Computational chemistry, Molecular dynamics, Statistical mechanics

## Abstract

We determined the effectiveness of absolute binding free energy (ABFE) calculations to refine the selection of active compounds in virtual compound screening, a setting where the more commonly used relative binding free energy approach is not readily applicable. To do this, we conducted baseline docking calculations of structurally diverse compounds in the DUD-E database for three targets, BACE1, CDK2 and thrombin, followed by ABFE calculations for compounds with high docking scores. The docking calculations alone achieved solid enrichment of active compounds over decoys. Encouragingly, the ABFE calculations then improved on this baseline. Analysis of the results emphasizes the importance of establishing high quality ligand poses as starting points for ABFE calculations, a nontrivial goal when processing a library of diverse compounds without informative co-crystal structures. Overall, our results suggest that ABFE calculations can play a valuable role in the drug discovery process.

## Introduction

The discovery of small molecules that bind a targeted protein with high affinity is a key early step in many drug discovery projects. This ligand-discovery step can take several years, and its cost on a per launched compound basis rivals that of later-stage clinical trials^1^. Various computational methods have therefore been developed that are aimed at speeding this step and lowering its cost. These methods include structure-based approaches, in which the three-dimensional structure of the targeted protein, usually obtained by X-ray crystallography, is used to guide the discovery of a tight-binding ligand. In one common paradigm, known as virtual compound screening^2^, a library of available candidate ligands is computationally docked^3^ to the targeted binding site, and compounds with favorable docking scores are procured and tested experimentally. Although such docking calculations can significantly enrich the yield of compounds that bind the targeted protein, i.e., of actives, relative to a random selection of compounds^4^, the accuracy of docking is limited by the simplifying approximations used to achieve high computational speed. For example, the protein is usually treated as largely rigid, solvent is treated rather crudely, and the entropic and energetic consequences of conformational fluctuations are handled implicitly at best.

In recent years, far more detailed computational methods of estimating protein-small molecule binding free energies that had been under development for decades have been widely adopted, due to their improved accuracy and the dramatic acceleration of simulations by the use of graphics processor units (GPUs)^5,6^. Most widely used are relative binding free energy (RBFE) calculations^7^, which yield the difference betweeen the binding free energies of two compounds by computing the free energy change of artificially, or alchemically, transforming one compound to the other in the binding site and in the bulk solvent^8^. The alchemical compound transformations in RBFE methods work particularly well when the two compounds being compared are chemically similar. As a consequence, RBFE methods are well suited for use in the hit-to-lead and lead-optimization stages of drug discovery^6^, because these stages typically involve explorations within a single congeneric series of compounds; i.e., a series of compounds that are chemically similar to each other. Good results have been obtained in retrospective benchmarks^7^ and in prospective industry applications^6^.

However, RBFE calculations can become difficult and even intractable when the two compounds of interest are chemically distinct from each other. One reason is that it can become difficult to design an alchemical pathway between the two compounds that affords good numerical convergence^9^. Another reason is that totally different compounds can adopt totally different orientations and conformations, or poses, in the binding site, and obtaining a correct result would require the molecular dynamics (MD) simulations to sample the interconversion of the two ligands’ poses. Such interconversions are usually very rare, at least when standard MD is used, because of the steric barrier to ligand motions imposed by the tightly fitted binding site. As a consequence, RBFE calculations are not as well suited to virtual compound screening, and thus are not available as a potential improvement over docking.

Absolute binding free energy (ABFE) methods^10^ are technically related to RBFE methods, but differ in that they directly yield the standard binding free energy of a given compound for the protein or receptor of interest. This quantity is computed from the reversible work of decoupling the ligand from the binding site and recoupling it with bulk solvent, in effect leaving the free ligand at standard 1 M concentration^11^. The decoupling and recoupling steps can be carried out by alchemical pathways^11–14^ or by physical pathways^15–19^. Importantly, ABFE methods can be applied directly to collections of compounds that are not chemically similar to each other. As a consequence, ABFE calculations might be suitable for virtual screening of diverse compounds^5,20^, except that they are much too slow to process an entire compound library. Thus, ABFE calculations are perhaps best used to refine initial docking results by providing more accurate discrimination between active and inactive compounds within focused sets of diverse compounds with high docking scores. However, a full-fledged test of whether ABFE can increase the enrichment of true actives relative to that afforded by docking, in the setting of virtual compound screening, has not yet been described, despite strong advances in ABFE implementations^20–28^. This delay may stem from the challenges associated with these calculations, such as protein conformational changes between bound and apo states, and the need to predict ligand binding poses^20,28,29^.

Here, building on our prior efforts to make ABFE calculations routine and efficient^20^, we evaluate the effectiveness of ABFE calculations as a tool to refine virtual screening calculations. Thus, we docked $\sim$ 70,000 active and decoy compounds across 3 protein targets, obtaining good initial enrichment of actives among the top-scored compounds. We then ran ABFE calculation on sets of high-scoring compounds for each target and found that the ABFE results differentiate true actives from decoys better than do the docking results. The present paper details the methods used and the results obtained and discusses potential implications and sources of error.

## Methods

### Overview of methods

Here, we first detail the proteins and ligands used in the study and how they were prepared for calculations. We then describe the computational approach used to compare ABFE calculations with docking in the context of structure-based virtual compound screening. In brief, for each target and its associated active and decoy compounds, we first used docking to rank all of the actives and decoys and identify the top-scoring form (protonation state, stereoisomer, tautomer) of each compound. Then, for two balanced sets of 30 compounds, ten docked poses of the top-scoring form were equilibrated in the binding site by MD, and any poses which moved away from the binding site were discarded. Full ABFE calculations were then run, starting from the best-scored remaining poses. The same ten poses were run twice through the full calculation, comprising MD equilibration and ABFE calculations, using different random number seeds, in order to generate two independent sets of results. These ABFE results are compared with corresponding docking results in terms of how well active compounds are enriched and differentiated from decoys.

### Protein-ligand systems and structure preparation

For protein targets BACE1, CDK2, and thrombin, all compound SMILES strings in the files actives_final.ism and decoys_final.ism were downloaded from the DUD-E website^30^. The actives are compounds with effective affinities of 1 μM or better, and the decoys are automatically generated compounds with molecular properties similar to those of the actives of the respective targets. (The DUD-E paper^30^ and website provide information on the physical properties of the active compounds.) These properties include molecular weight, water-octanol partition coefficient, number of rotatable bonds, numbers of hydrogen bond acceptors and donors, and net charge. However, the decoys are quite different in chemical structure from the actives, so the decoys are unlikely to be active against the target^30^. Targets BACE1, CDK2, and thrombin are associated respectively with 283, 474, 461 actives and 18100, 27850, and 27004 decoys. The compound SMILES strings were imported to Maestro^31^ and processed using Ligprep^31,32^. In order to account for possible changes in protonation and tautomer states on binding, candidate alternate protonation and tautomer states were generated, along with an Epik penalty term, a quantitative estimate of the relative stability of each form. In addition, alternate stereoisomers were generated for compounds with undefined stereocenters in their SMILES strings. We assumed that such compounds were racemic mixtures, so the affinity of the stereoisomer which binds the best should be a good approximation to the binding affinity of the mixture. The candidate protonation states were generated by modeling a range of pH values centered approximately on the pH at which the experimental binding measurements were executed. For CDK2, candidate protonation states were generated for the pH range 5 to 9. For thrombin, candidate protonation states were generated for the pH range 5.5 to 9.5. For BACE1, ligand protonation states were generated for pH values down to 0, to account for the lower experimental pH of 4.5; and up to 10, to allow for the possibility of relatively large upward shifts of ligand pKa due to the two charged aspartates in the catalytic site.

For each protein target, a ligand-bound co-crystal structure with high quality metrics^33^ was imported from the PDB^34^ to Maestro and processed with the Protein Preparation Wizard^31,32^. For BACE1, a monomeric unit from PDB entry 6UWP was kept, and the protein was protonated for pH 4.5. For CDK2, the kinase and its bound cyclin from PDB entry 3DDQ were kept and were protonated for pH 7.0. For thrombin, a unit containing the heavy chain, the light chain, hirudin, and the bound ions from PDB entry 5JZY were kept and protonated for pH 7.5. These pH values were chosen to be typical of those used in the affinity assays for each respective protein^35–37^. For all targets, crystal waters were retained during structure preparation.

### Protein-ligand docking

The prepared protein structures were used to generate receptor grids for docking with Glide SP^31^. (Test calculations using Glide XP instead of Glide SP gave somewhat worse enrichment statistics.) Hydroxyls near the binding site were set as rotatable. Crystal waters that had been included during structure preparation were removed for grid generation and docking. The Glide option of 4x enhanced conformational sampling was used, but other Glide options were kept at their defaults. Every candidate chemical form (protonation states, tautomers, stereoisomers) of each ligand was docked and the Epik protonation state penalty was incorporated into the docking score. The penalty relates to the estimated pKa of each protonatable moiety and the pH at which the target’s binding assay was carried out. The final score for each compound was taken to be that of the best-scoring pose across all candidate chemical forms of the compound. We assessed docking performance by using Maestro’s enrichment calculator to determine the 1% enrichment factor and the area under the receiver operating curve (AUC) across all actives and decoys for each target, and compared these results with those previously obtained for these datasets in a prior study^30^. Given a set of *N* ligands of which $N_A$ are known actives, if $n_A$ actives are in the top 1% of compounds by docking score, then the 1% enrichment factor is $\frac{n_A}{0.01 N}/\frac{N_A}{N}$.

### Selection of compounds for ABFE calculations

For each protein target, two compound sets, each containing 30 compounds, were selected based on the docking results. The Tier 1 compounds comprise the 30 highest scoring compounds following downsampling of all actives by a factor of two for BACE1 and three for CDK2 and thrombin. Downsampling the actives reduces the statistical error of the AUC by making the number of Tier 1 actives about equal to the number of Tier 1 decoys^38^). The Tier 2 set for each target similarly contains 15 actives and 15 decoys randomly drawn from compounds in a slightly less favorable range of docking score: -8.0 to -7.0 for BACE1 and -9.8 to -8.0 for CDK2 and thrombin. No other criteria were considered when drawing these compound sets. The Tier 1 set resembles a set of the most promising compounds in a virtual screening scenario, when docking score is the only screening criterion. The Tier 2 set is also relevant because in drug discovery, compounds are usually triaged not solely based on docking score, and because docking is meant to be effective in enrichment but not necessarily effective in predicting affinity ranking. Maestro’s enrichment calculator was again used when assessing docking and ABFE on these compound subsets.

### MD equilibration

To establish starting conformations for the MD-based ABFE calculations, ten distinct docked poses of the top-scoring chemical form for each compound in the Tier 1 and Tier 2 compound sets for each target were generated with the docking procedures described above. To make poses meaningfully different from each other, a new pose was accepted only when its root-mean-square deviations (RMSD) from all other poses were $> 2$Å or if any atom was $>5$Å from the same atom in all other poses. The ten poses usually have docking scores ranging across a few units, e.g, from − 9 for the most favorable pose to − 6 for the least favorable pose. Although these docked poses could in principle be used directly as starting points for the ABFE calculations, our pilot studies indicated that this yielded relatively poor results. We therefore pre-processed each pose with a short MD simulation of the protein-ligand complex using the same force field and explicit solvent as the subsequent free energy calculation (Section “Absolute binding free energy calculation”), thus allowing the system to equilibrate before the production calculations. In some cases, a docked pose was not stable, and the ligand moved away from the binding site during equilibration. This happens more often for poses with worse docking scores, though the correlation is by no means perfect. Such poses were not advanced to the free energy simulation stage. Details of this MD equilibration step follow.

The program BAT.py version 2.0^20^ was used to prepare the equilibration simulations, which were run with the simulation engine AMBER^39^. This is referred to as the equil stage in BAT.py. The simulations used the exact same prepared protein-ligand system as did the docking calculations, except that crystallographic waters which do not clash with any ligand in the compound set were now included. The protein with its docked ligand was exported from Maestro and parsed into AMBER files to build the simulation system. In keeping with our previous ABFE study^20^, we used the AMBER ff14SB force field for the protein, GAFF version 1^40^ with AM1/BCC charges^41^ for the ligand, and the TIP3P model^42^ for water molecules. Bulk water molecules were added to form a cubic solvent box in a manner that ensured distances > 20 Å between the surfaces of the protein and its periodic images throughout the simulations. Sodium or chloride ions were added to neutralize the simulation system. The resulting system was energy-minimized, and translational and rotation restraints of the ligand relative to the receptor were applied, using the same scheme and force constants as for the subsequent ABFE calculations (Section “Absolute binding free energy calculation”). MD simulations, with a time step of 4 fs (made possible by our use of hydrogen mass repartitioning^43^), temperature control via Langevin dynamics^44^, and Monte Carlo barostat, were then carried out in four stages: heating to 298 K over 0.1 ns, NPT equilibration over 0.4 ns, gradual release of ligand restraints over 0.4ns, and finally a 12 ns MD simulation with Monte Carlo/MD exchange of waters near the ligand^45^. The temporary application of ligand restraints allows the protein to relax somewhat around the ligand in its docked pose before the ligand is given the freedom to potentially drift away.

The coordinates of the last simulation frame were used as starting points for the subsequent stage of the ABFE calculation, unless the ligand had moved too far from the binding site’s key residues, indicating an unstable pose. In particular, a pose was considered unstable if, in the final snapshot of the MD equilibration simulation, no ligand atom was within 4.5 Å of a selected atom in the parent co-crystal structure. For BACE1, this is the nitrogen of the 6WUP ligand that is closest to the two catalytic Asp residues. For CDK2, this is the nitrogen of the 3DDQ ligand closest to the molecular fork^46^ formed by Glu81 and Leu83. For thrombin, this is the amide nitrogen of the 5JZY ligand near His57 of thrombin’s catalytic triad. The best-scoring 5 poses that passed the filter were sent to the next stage, free energy simulation.

### Absolute binding free energy calculation

We next applied the BAT.py binding free energy script to each of the five poses from the above MD equilibration step, using the simultaneous decoupling and recoupling approach^20^. This calculation involves computing the following free energy components: attachment of receptor conformational restraints with ligand in binding site; attachment of conformational restraints to the bound ligand; attachment of translational and rotational restraints to the bound ligand; simultaneous decoupling and recoupling of ligand charge interactions; simultaneous decoupling and recoupling (SDR) of ligand LJ interactions; release of ligand translational and rotational restraints for the ligand in bulk solvent, leaving ligand freely rotating and effectively at standard concentration; release of ligand conformational restraints in bulk solvent; and release of receptor conformational restraints for the receptor without the bound ligand. The conformational restraints comprise harmonic distance restraints among three protein anchor atoms and three ligand anchor atoms; for details, see the BAT.py user manual and provided input files. Note that the SDR method^20^ does not remove the ligand from the simulation box and therefore does not cause a change in net charge of the system for charged ligands, so no special procedures are required for charged ligands.

For each component, a series of 10 to 16 independent parallel simulation windows, covering the range of restraint weights or transformation lambda values, were run and were analyzed by MBAR^47^ or thermodynamic integration, except that the free energy of releasing the ligand translational and rotational restraints was evaluated analytically^20^. Summing the free energy contributions from each component gives the ABFE of the ligand in the simulated pose. The same simulation procedures were applied independently to each pose generated by the MD equilibration stage described above, giving five binding free energy results for each compound, and the overall binding free energy of the compound was obtained by combining the result for all five poses according to Eq. (1)^17,20^.

1 $$\begin{aligned} \Delta G^\circ _\text {overall} = - RT \ln \sum _{i}^{N_\text {poses}} e^{-\beta \Delta G^\circ _i} \end{aligned}$$

Note that the pose with the most favorable (negative) binding free energy contributes the most to the overall binding free energy. The numerical uncertainty due to finite simulation time during the free energy runs was estimated by five-block blocking analysis for each pose and propagated through Eq. (1) to obtain the uncertainty of the overall binding free energy, using the python package Uncertainties of Eric O. Lebigot. We report metrics of performance (e.g. area under the receiver operating characteristic curve) based on the more favorable overall binding free energy from the two independent runs for each ligand.

These free energy simulations used the same force field parameters and core simulation settings (e.g., time step, temperature, pressure, treatment of short- and long-ranged nonbonded interactions) as the MD equilibration stage described above. Each window ran for 1 to 3ns of simulated time, with about two thirds of the MD time corresponding to production stage simulations whose frames enter the free energy analysis. Further details of the BAT.py procedures can be found in a previous publication^20^ and on the BAT.py Github page. Using RTX3090 GPU yielding $\sim$ 300 ns/day of simulated time for the solvated systems in this study, the free energy simulations can be completed in about 2 h wall time when running in parallel with dozens of GPU, or in about 1 day per pose when running sequentially with single GPU. This free energy stage is the computational bottleneck in the overall procedure, because the MD equilibration stage and docking are much cheaper computationally.

## Results and discussion

We evaluated ABFE as a virtual screening tool based on data for the protein targets BACE1, CDK2, and thrombin. For each target, we docked and scored a mixture of several hundred known actives and 18,000–28,000 presumed inactives (decoys) drawn from the DUD-E resource. We then compared the ability of docking and ABFE to distinguish actives from decoys among tractable sets of compounds with the very best docking scores (Tier 1 compounds) and among compounds with somewhat worse docking scores (Tier 2 compounds). In order to put these results into context, we also compared the accuracy of our docking calculations across all compounds with those previously reported in the original DUD-E paper^30^. This section presents these ABFE and docking results and discusses factors that limit ABFE precision and accuracy and how these factors suggest future directions to improve the ABFE approach.

### Enrichment of known actives by ABFE

**Figure 1:**
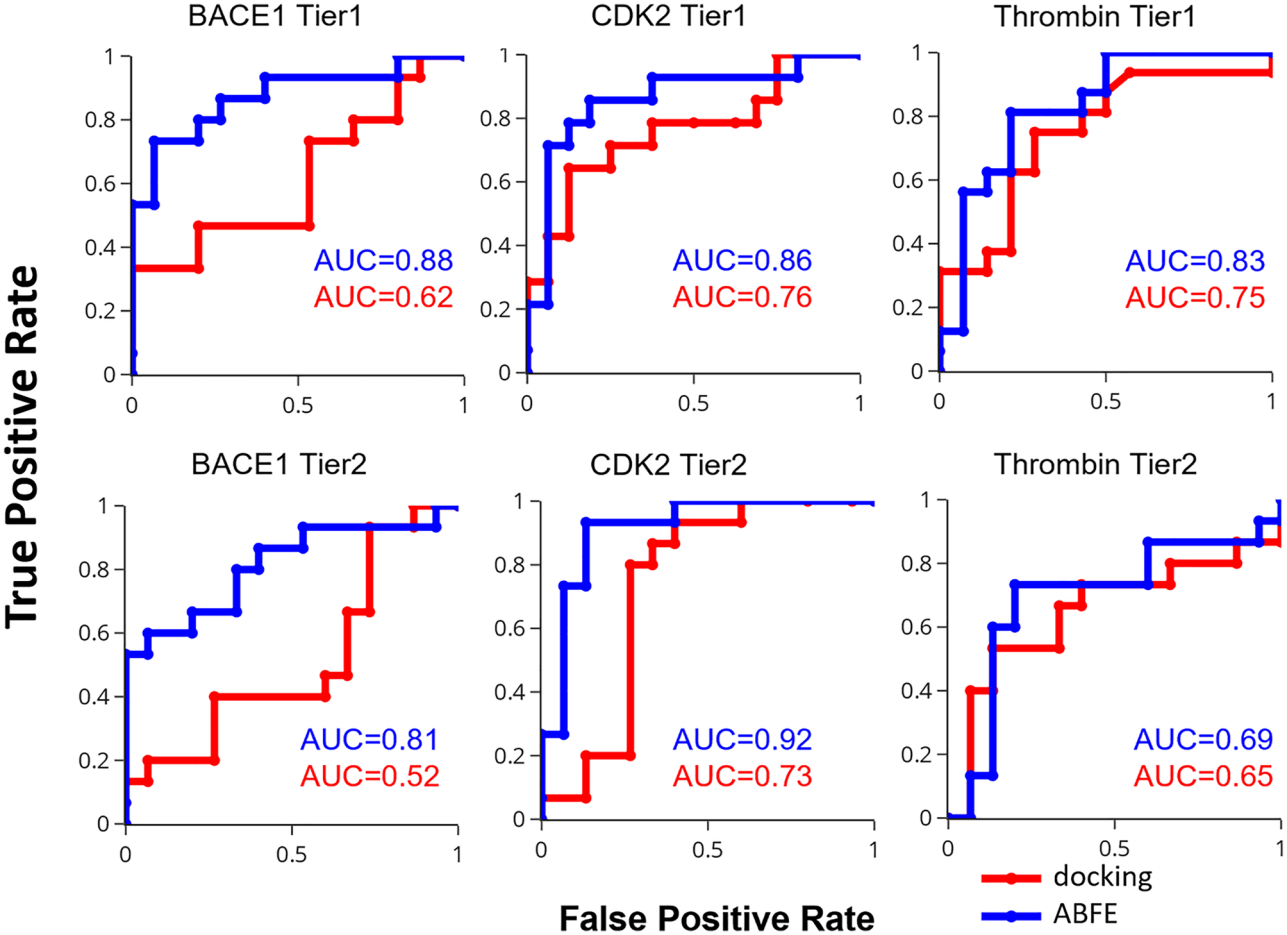
Comparison of the ability of ABFE calculations versus docking to distinguish active compounds from inactives (decoys), shown as Receiver Operating Characteristic (ROC) curves with the Area Under Curve (AUC) statistics for the 30 Tier 1 and 30 Tier 2 compounds of all three protein targets, as labeled. Red: docking results. Blue: ABFE results. These ABFE calculations omit the free energy term for ligand protonation state changes that were incorporated into the docking calculation. However, adding this term to the ABFE results leads to negligible changes in the AUC statistics (maximum change 0.02, mean change 0.00).

**Figure 2:**
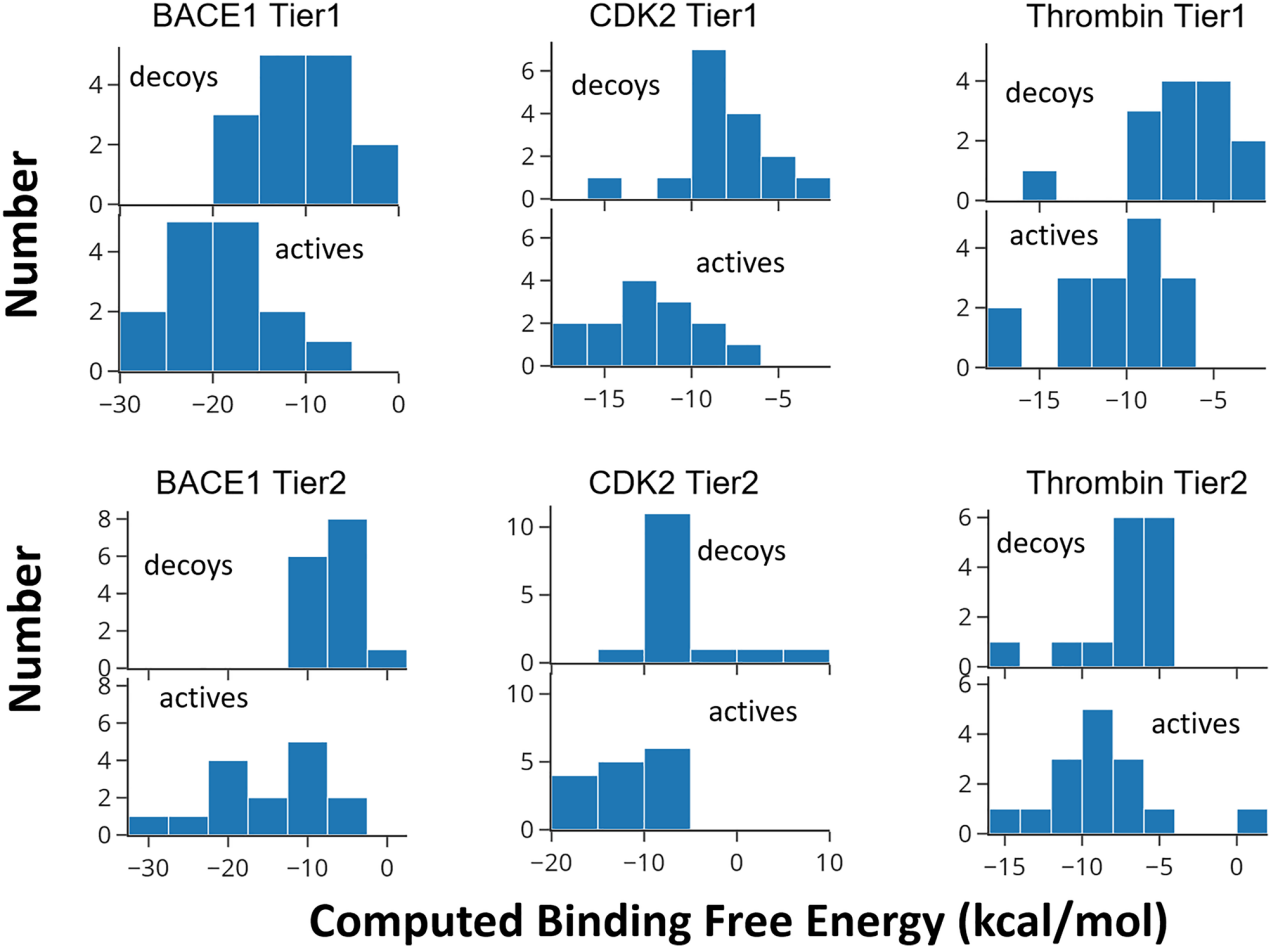
Comparison of distributions of computed ABFE values for inactive (decoy) and active compounds for Tier 1 and Tier 2 of all three protein targets, as labeled.

The present ABFE calculations outperform docking calculations for both Tiers of ligands on all three targets, as assessed from the areas under the curve of their respective receiver-operating characteristic curves (Fig. 1). Across all six tests—three targets, each with two tiers—the AUC increases by an average of 0.16 (0.67 to 0.83) on going from docking to ABFE, with BACE1 improving most and thrombin least. Thus, a central conclusion of this work is that ABFE calculations can yield greater enrichment of actives over decoys in the context of virtual compound screening.

Histograms of the computed ABFEs for the actives and decoys in all six tests detail the negative (favorable) displacement of the actives’ ABFE distributions relative to those of the decoys (Fig. 2). The analogous plots for docking scores in SI show smaller separations between the actives and decoys. However, there is still significant overlap between the two ABFE distributions in all six cases, as some decoys are assigned quite favorable binding free energies.

### Baseline enrichment of known actives by docking

Our observation that ABFE calculations outperform docking in virtual compound screening would not be very meaningful if we were comparing it with an unreliable docking method. However, our docking calculations do yield good enrichment metrics for the focused Tier 1 and Tier 2 sets, as assessed by the AUC statistic (Table 1). Thus, our docking AUCs range from 0.82 to 0.87, and our 1% enrichment factors range from 26 to 30. Indeed, compared with the original DUD-E paper^30^), the present docking calculations over each target’s full DUD-E dataset of 18,000–28,000 compounds achieve substantially better enrichment metrics for BACE1 and CDK2, and similar metrics for thrombin (Table 1). These favorable results for our baseline docking method indicate that the enhanced differentiation of actives from decoys afforded by ABFE represents a meaningful advance. It also suggests that the docked poses generate by our docking procedure are suitable as starting conformations for downstream ABFE calculations.

Our improved docking results may stem in part from our choice of target protein structures^33^ and/or our use of the Glide SP method. However, for BACE1, the enhanced docking enrichment observed here appears to result more from our treatment of protonation states, because our initial docking experiments with Glide SP but without these refined protonation state protocols gave worse BACE1 enrichment metrics, similar to those from the prior study. First, despite our selecting a pH of 4.5 typical of BACE1 binding assays^35^, Maestro assigned both of the active site aspartic acids, which are critical to binding, as ionized. Although such a low pH might be expected to cause at least one of the aspartic acids to be predominantly neutral, we accepted the Maestro assignments based on prior computational studies of this system^48,49^. Second, we used LigPrep to generate all ligand protonation states predicted to be plausible for the free ligand over the wide 0 to 10 pH range, instead of the narrower 6 to 8 pH range in the previous study. We then docked all states and chose the state with the best docking score, accounting for any protonation free energy penalty. This approach may be particularly relevant for BACE1, with its two ionized aspartic groups in the binding site, which may shift the ligand to more protonated states.

**Table 1:** Enrichment of actives over decoys by docking in previous^30^ and current study of these target/compound sets, as measured by the Area Under Curve (AUC) of the ROC curve (Receiver Operating Characteristic curve) and the enrichment factor of the top scoring 1% compounds. Note that these statistics are computed for the full set of $\sim 20,000$ compounds per target, while those in Fig. 1 are for the Tier 1 and Tier 2 subsets.

	BACE1		CDK2		Thrombin	
	Previous	Current	Previous	Current	Previous	Current
AUC of ROC curve	0.66	0.85	0.79	0.87	0.81	0.82
1% Enrichment factor	8	28	14	30	30	26

**Table 2:** ABFE results for the BACE1 Tier 1 compound set. Compound: nomenclature as in^30^; actives begin with CHEMBL. For each compound, both the more favorable and the less favorable overall binding free energies (BFE, kcal/mol) from the two independent runs are shown. Blocking uncertainties (kcal/mol) and pose-specific ABFE values (kcal/mol) are also presented for the more favorable result. Diff: difference between the two overall BFE values. Docking: docking score computed in this study.

Compound	More favorable BFE run			Less favorable BFE run	Diff	Docking
	Overall BFE	Blocking uncertainty	Pose BFEs	Overall BFE		
**CHEMBL257091**	− 27.1	1.5	− 27.1, − 3.9, − 10.8, − 4.0, − 4.0	− 21	6.1	− 8.8
**CHEMBL502121**	− 25.1	2	− 13.4, − 8.7, − 25.1, − 14.6, − 6.5	− 19.1	6.0	− 9.4
**CHEMBL260834**	− 22.8	1.8	− 12.6, − 22.8, − 13.0, − 13.1,4.9	− 17.9	4.9	− 8.7
**CHEMBL502289**	− 22.6	1.2	− 22.2,0.4, − 9.0, − 1.4, − 22.2	− 22.3	0.3	− 9.2
**CHEMBL1092147**	− 21.9	1.1	− 19.4, − 21.9, − 1.7,4.9, − 14.9	− 17	4.9	− 8.7
**CHEMBL230245**	− 21.3	1.9	− 14.9, − 17.8, − 10.2, − 21.3, − 12.8	− 19	2.3	− 9
**CHEMBL595065**	− 21.1	2.1	− 18.5, − 17.4, − 20.6,6.5, − 20.8	− 18.8	2.3	− 8.5
**CHEMBL571433**	− 19.8	3	− 4.1, − 19.8, − 10.2, − 1.7, − 7.3	− 10.9	8.9	− 9.6
**C01491960**	− 18.2	1.7	− 8.3, − 10.3, − 18.1, − 3.4, − 16.9	− 13.7	4.5	− 8.8
**CHEMBL404839**	− 18.1	1.8	− 8.4, − 18.1, − 12.2, − 13.9, − 15.3	− 18.1	0.0	− 9.3
**CHEMBL257645**	− 17.1	1.5	− 9.0, − 8.2, − 17.1, − 14.9, − 0.0	− 15.9	1.2	− 8.7
**CHEMBL500555**	− 17	2.5	− 17.0, − 9.4, − 10.6,3.5, − 9.2	− 17	0.0	− 8.6
**C39631886**	− 16.5	1.9	− 6.1, − 16.5, − 11.5, − 5.5, − 7.8	− 12.5	4.0	− 8.5
**C39559755**	− 15.4	1.3	− 5.9,5.2, − 15.4, − 7.5, − 4.0	− 11.7	3.7	− 8.5
**CHEMBL1092146**	− 15.3	1.9	− 13.2, − 15.3, − 12.6,1.9, − 0.2	− 14.7	0.6	− 8.6
**C22874288**	− 14.2	1.2	− 14.2, − 3.4,0.9, − 5.8, − 2.1	− 9.6	4.6	− 8.8
**CHEMBL595066**	− 14	1.3	− 14.0, − 10.2, − 2.1, − 7.8,1.2	− 13.5	0.5	− 8.6
**C39674030**	− 12.1	2	− 3.9,1.3, − 12.1, − 10.0,0.5	− 11.6	0.5	− 8.8
**C28524322**	− 11.8	1.7	− 11.8, − 5.7, − 6.7, − 3.9, − 4.6	− 11.6	0.2	− 8.7
**CHEMBL517179**	− 11.4	1.4	− 11.4, − 4.5, − 3.4, − 7.7, − 0.6	− 9.7	1.7	− 8.9
**C39631541**	− 10.4	1.8	− 3.9, − 10.4,2.8, − 6.2,4.6	− 9	1.4	− 8.7
**C28706109**	− 10	1.6	− 5.7,6.4, − 5.8, − 10.0, − 5.2	− 5.8	4.2	− 9
**C39674755**	− 10	2.4	8.4, − 1.3,1.0,0.3, − 10.0	− 6.4	3.6	− 8.6
**C39669490**	− 8.9	1.1	− 2.9, − 8.6, − 2.6, − 6.8, − 8.2	− 5	3.9	− 8.6
**C35048276**	− 8.6	1.4	− 1.1, − 8.6, − 3.7, − 3.1, − 3.9	− 6.7	1.9	− 8.5
**C35750025**	− 7	2.1	− 7.0,6.3,2.4,1.6,2.9	− 5.6	1.4	− 8.8
**CHEMBL1092788**	− 6.4	0.9	− 4.9, − 6.0, − 5.8, − 1.8, − 4.8	− 6	0.4	− 8.7
**C40318643**	− 5	1.3	− 3.5, − 2.0, − 0.8, − 2.8, − 5.0	− 4.7	0.3	− 8.9
**C27260756**	− 4.8	1.6	0.8, − 4.8, − 0.7, − 1.0, − 1.5	− 2.6	2.2	− 8.9
**C36064029**	− 4.7	2.4	0.8,2.2, − 2.4,0.2, − 4.7	− 4.1	0.6	− 8.8

### Analysis of errors

#### Overly favorable computed binding affinities

As noted above, the ABFE calculations predict that some decoy compounds have quite favorable binding free energies. Some of these DUD-E decoys may truly be active^30^, especially as our docking calculations should have enriched the fraction of true actives among the high-ranking decoys studied here. However, we believe the apparent overestimation of many decoy binding affinities reflects imperfections in the ABFE calculations, particularly given that the ABFE calculations also predict excessively favorable binding affinities for some actives, as evident in Fig. S2. Two possible explanations of this problem come to mind. The first is that errors in the force field may cause these affinities to be overestimated. If so, this would most likely result from an imbalance in nonbonded interactions, i.e., in the electrostatic and Lennard-Jones terms of the protein, the ligand, and/or the aqueous solvent. Interestingly, two lines of evidence suggest that standard water models lead to overestimation of effective intramolecular protein attractions^50^ and host-guest attractions^51^ and have motivated adjustments to the TIP4P^50^ and TIP3P^51^ models, respectively. It is possible that the same imbalance leads here to overestimation and that these modified water models would reduce or abolish the apparent overestimations observed here. Also, given the diversity of ligand chemical structures, bespoke ligand force field parameters optimized separately for each ligand may model the ligand conformational landscape more accurately^6,52–54^. The second possible explanation is that our ABFE calculations may not adequately capture the fall in free energy associated with relaxation of the ligand-bound protein to its unbound conformational ensemble following decoupling of the bound ligand^29^. Underestimating this fall would cause the free energy of dissociation to appear less favorable than it ought and therefore make the binding free energy overly favorable.

#### Lack of correlation between computed and experimental binding free energies

Although the present ABFE calculations successfully enhance the enrichment of known actives from decoys, we find essentially no correlation between our computed ABFEs and the available affinity data for the actives, as provided in the DUD-E dataset used here (Fig. S2). This contrasts with prior ABFE studies which, using similar force fields, have obtained significant correlations between calculation and experiment^28,55,56^. We believe this difference results primarily from differences in the accuracy of the ligand poses used to initiate the ABFE calculations. Here, we have deliberately replicated a virtual screening setting where there is minimal prior information about the poses of the chemically diverse ligands to be screened. Accordingly, our initial poses were generated by unsupervised docking into a single protein crystal structure for each protein target. Although we used a reasonable docking protocol, docking is still far from exact, and the imperfections and inconsistencies in our starting poses undoubtedly contributed to the errors in our ABFE results.

In contrast, prior tests of ABFE methods appear to have utilized relevant structural information to obtain relatively refined poses not available in the setting of a real world virtual screening campaign. In particular, some studies have applied their ABFE methods to congeneric series of ligands, where a consistent set of starting poses could be generated by overlaying the compounds’ common chemical scaffold on that observed in a co-crystal structure for one of the compounds in the series^28,29,55–57^; and other studies considered chemically diverse ligands but started each ABFE calculation from the available co-crystal structures of the respective ligand^55,56^.

In conclusion, although successful virtual screening does not require a high correlation between computed and experimental affinities among true binders, docking methods that give more reliable pose predictions for diverse compounds would likely lead to futher improvement of virtual screening via ABFE calculations.

#### Reproducibility of ABFE results

**Figure 3:**
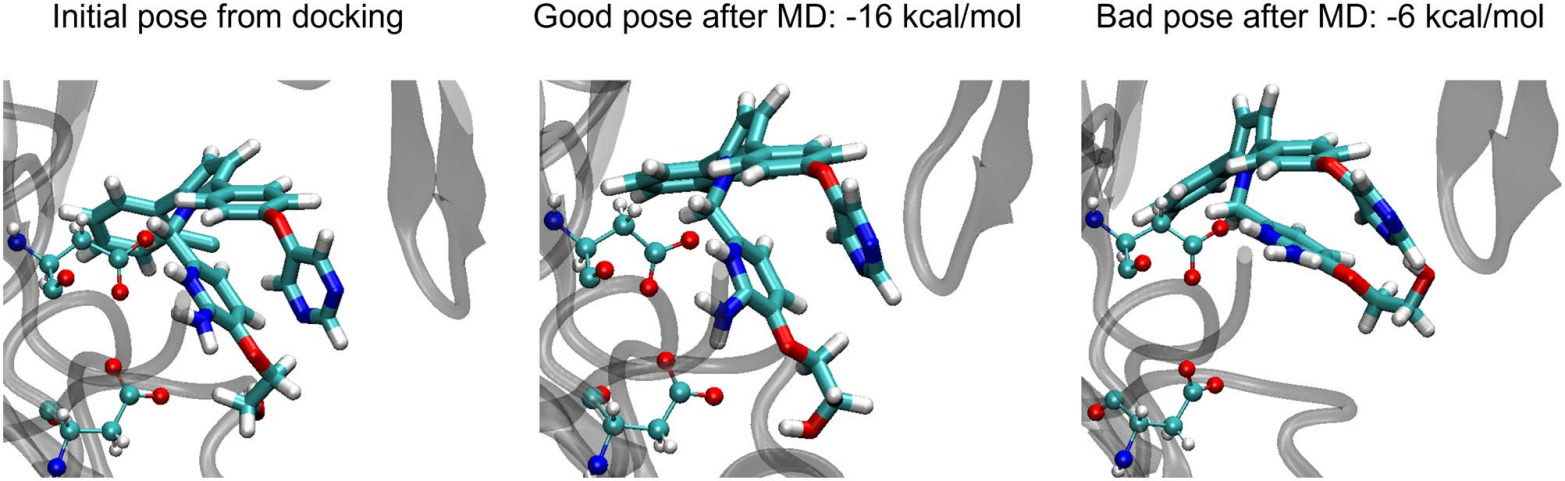
Initial ligand conformations from docking and for the two independent runs of BACE1 active compound CHEMBL1090542. The same initial ligand pose (left) from docking relaxes to two different conformations during the MD equilibration step. In the run that yields the more favorable BFE (middle), the ligand stays close to the initial pose, whereas in the less favorable run (right) the ligand drifts away and loses interaction with the catalytic Asp residues, resulting in much less favorable binding free energy. BACE1 is shown in ribbon representation, the two catalytic Asp residues in ball and stick representation, and the ligand in licorice representation.

Given a set of initial docked poses, the precision of our ABFE calculations is affected by two levels of numerical uncertainty. One, the numerical convergence of the free energy calculations as a function of simulation time, is estimated by blocking analysis to be about 0.5 to 3 kcal/mol (Table 2 and SI Tables). This uncertainty could be reduced by using longer MD runs. The other, larger, source of uncertainty is evident from the differences between the two independent ABFE runs started from the same set of docked poses, one of which yielded the more favorable BFEs considered in the prior subsections, the other of which yielded the less favorable BFE as shown in Table 2 and SI tables. The differences between these two runs range from 0 to about 9 kcal/mol. Detailed examination of cases where this deviation is large reveal that they occur when the MD equilibration stages of the two independent runs, which are initiated from the same docked pose, lead to significantly different post-equilibration conformations. The ligand conformational restraints routinely applied at the next stage of the ABFE calculation can lock in this conformational difference, leading to significantly different ABFE results. For example, the same initial ligand pose from docking of the BACE1 ligand CHEMBL1090542 relaxes to two quite different conformations in the two independent MD equilibration phases. In one run, the ligand conformation stays close to the initial pose from docking (Fig. 3, left and middle panels), resulting in the more favorable ABFE of -16 kcal/mol. In the other run, the ligand loses its initial close interaction with the catalytic Asp residues (Fig. 3 right panel), resulting in the less favorable binding free energy of -6 kcal/mol. Note that similar problems can occur if the receptor makes a conformational transition that equilibrates slowly relative to the simulation time.

We currently alleviate this sampling problem by doing two independent ABFE calculations for the same docked pose and keeping the more favorable of the two resulting BFEs, since the tightest-binding post-equilibration pose is the more stable one and hence presumably more realistic. Additional independent runs would further increase the possibility of including the best post-equilibration ligand conformation, but this would come at a cost of lower computation throughput. It is also worth noting that the relatively large differences between independent ABFE calculations helps motivate our use of relatively short simulation window lengths: longer windows would slow the calculations without reducing the chief source of numerical uncertainty.

One might expect that initiating ABFE calculations directly from docked poses—i.e., skipping the MD equilibration step—would solve this problem, but in practice it led to worse AUC statistics when we tried it. This may result from incompatibility between the simplifications made in docking and the more detailed description used in explicit-solvent MD simulations, or from differences between the OPLS2005 ligand force field used in Glide docking and the GAFF ligand force field used in the MD simulations.A more effective approach might be to devise a fast method of estimating the relative stabilities of multiple poses discovered during the MD equilibration stage.

## Conclusions

This study demonstrates that integrating ABFE calculations into structure-based virtual compound screening yields more accurate discrimination between active and inactive compounds than docking alone. This approach can be used as is to speed early-stage drug discovery. The study also provides insights into errors associated with such calculations and thus suggests directions for future improvements within this broad approach.

## Supplementary Information

Supplementary Information.

## Data Availability

Input files used in this study, such as protein coordinate files, can be accessed at github.com/fengmudong/ABFE-paper.
