# Supplementary material for: Absolute binding free energy calculations improve enrichment of actives in virtual compound screening
Source: Sci Rep. 2022 Aug 10;12:13640. doi: 10.1038/s41598-022-17480-w (PMC9365818; doi:10.1038/s41598-022-17480-w)
Supplement: Supplementary file 1 — Supplementary Information. [file 41598_2022_17480_MOESM1_ESM.pdf]

Supplementary Information for "absolute binding free energy calculations improve enrichment of actives in virtual compound screening"

Mudong Feng[1]**, Germano Heinzelmann**[2]**, and Michael K. Gilson**[1,*]

[1]Department of Chemistry and Biochemistry, and Skaggs School of Pharmacy and Pharmaceutical Sciences, UC San Diego, La Jolla, CA 92093, USA
[2]Departamento de Física, Universidade Federal de Santa Catarina, Florianópolis, Santa Catarina, Brazil
[*]mgilson@health.ucsd.edu

Input files used in this study, such as protein coordinate files, can be accessed at github.com/fengmudong/ABFE-paper.

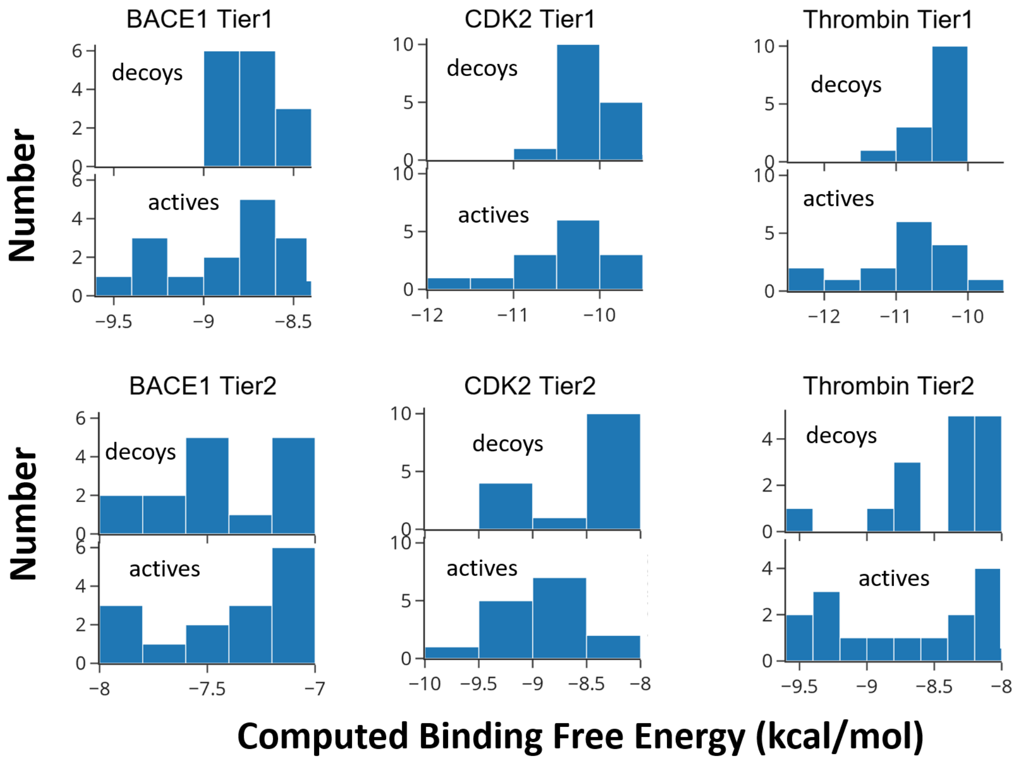

Figure S1. Comparison of distributions of computed docking scores for inactive (decoy) and active compounds for Tier 1 and Tier 2 of all three protein targets, as labeled.

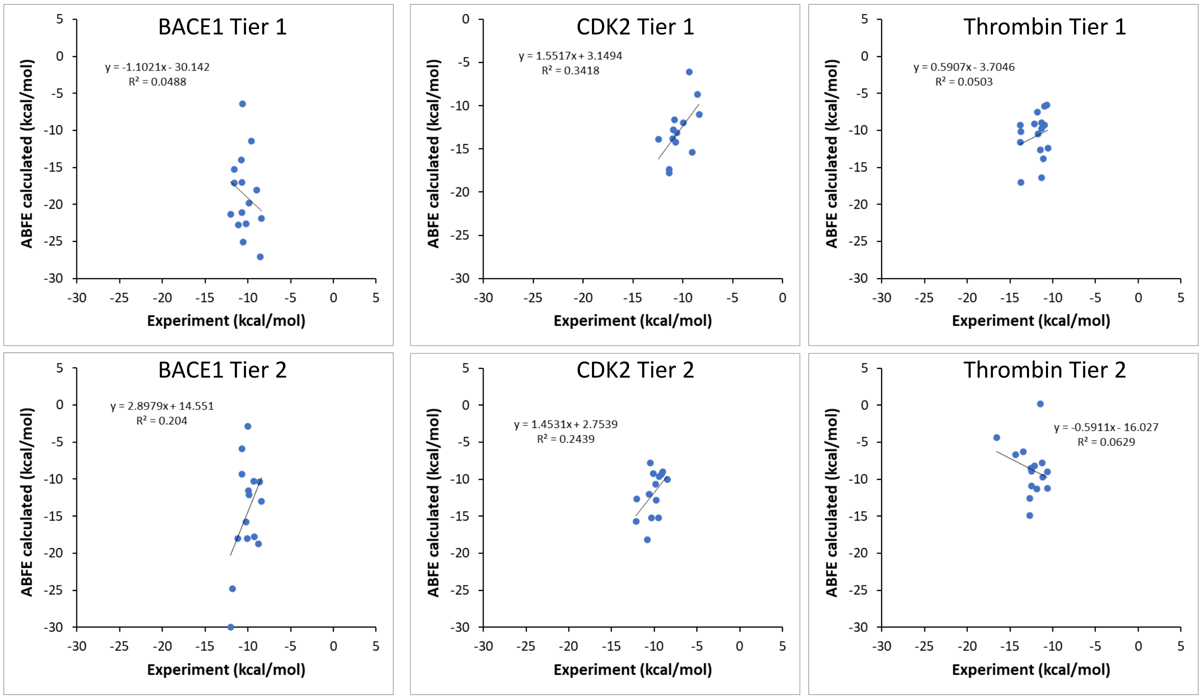

Figure S2. Scatter plots of computed absolute binding free energy against estimated binding free energy for Tier 1 and Tier 2 of all three protein targets, as labeled. Experimental binding free energies are estimated as $-RT\ln C_{50}$, where C_{50} is the measured IC50 or dissociation constant (K_D).

	More Favorable BFE Run			Less Favorable BFE Run		
	**Overall BFE**	**Blocking Uncertainty**	**Pose BFEs**	**Overall BFE**	**Diff**	**Docking**
**CHEMBL539436**	-30	2.1	-3.1, -5.0, -30.0, -27.5, -18.8	-25.1	4.9	-7.6
**CHEMBL403727**	-24.8	1.9	-24.8, -17.0, 7.4, 1.5, -1.9	-14.9	9.9	-8
**CHEMBL494186**	-18.7	1.5	-9.1, -16.2, -8.4, -18.7, -1.2	-13.4	5.3	-7.2
**CHEMBL244347**	-18	1.6	-18.0, -2.0, 1.1, -7.7, -14.1	-6.4	11.6	-7.2
**CHEMBL1162068**	-18	1.8	-17.6, -17.5, -12.1, -11.3, -7.6	-17.4	0.6	-7.3
**CHEMBL589614**	-17.8	0.8	-11.5, -13.8, -17.8, -5.0, -15.3	-17.7	0.1	-7.2
**CHEMBL1090542**	-15.8	1.4	-15.7, -10.3, -4.4, -14.2, -6.2	-14.6	1.2	-7.9
**CHEMBL1091343**	-13	1	-8.6, -13.0, -10.0, -6.1, -7.7	-12.8	0.2	-7.2
**C36748732**	-12.2	1.3	-12.2, 5.1, 1.4, 2.2, 1.3	-11.4	0.8	-7.1
**CHEMBL566638**	-12.1	1.4	-6.5, -12.1, -6.6, 2.7, -0.5	-7.5	4.6	-7.3
**C39654293**	-11.8	1.6	-6.5, -8.1, -11.8, -0.1, -9.7	-9.8	2	-7.5
**C39664437**	-11.7	1.2	4.1, -2.8, 9.2, -4.5, -11.7	-7.2	4.5	-7.9
**CHEMBL582982**	-11.6	1.2	-11.6, 0.5, 1.5, -1.7, -4.6	-8.4	3.2	-7.6
**C39685692**	-10.9	2.2	-7.7, -0.8, -2.4, -10.9, -7.2	-9.9	1	-7.9
**C39672053**	-10.6	1.6	-2.5, -10.6, 0.1, 0.8, -6.8	-6.9	3.7	-7.5
**CHEMBL1209286**	-10.4	3	-10.3, 3.7, 0.6, 0.6, -2.7	-4.9	5.5	-7.2
**CHEMBL1209105**	-10.3	1.3	-10.3, -2.5, -2.8, -1.6, 7.3	-5.6	4.7	-7.1
**C39666396**	-10	1.8	4.7, 6.0, -10.0, 0.7, 4.1	-8.8	1.2	-7.1
**CHEMBL1082548**	-9.3	2.6	-3.3, -3.6, 0.9, 2.7, -9.3	-5.8	3.5	-7.1
**C06282188**	-7.5	1.9	-7.5, 0.8, 4.6, -2.4, 8.2	-5.8	1.7	-7.3
**C09972247**	-6.4	0.9	-6.4, -3.9, -3.0, 2.0, 5.8	-2.1	4.3	-7.5
**CHEMBL570298**	-5.9	1.2	-5.3, -5.7, 0.4, 2.0, -0.6	-5.2	0.7	-7.6
**C20289590**	-5.1	3	-5.1, 6.0, 4.0, 0.6, 11.1	-0.5	4.6	-7.5
**C19833178**	-5	0.7	-5.0, -0.9, -1.9, 5.2, -1.1	-2.9	2.1	-7.7
**C20191597**	-4.4	1.3	-3.8, -4.1, -1.5, 3.7, 6.4	-4.1	0.3	-7.7
**C22691364**	-4.3	1.7	0.5, -1.8, -4.3, 0.0, 6.8	-1	3.3	-7.2
**C35939451**	-3	1.8	0.3, 5.4, 0.4, 14.1, -3.0	-1.8	1.2	-7
**C08820594**	-3	1.1	-1.9, 2.0, -1.7, nan, -2.8	-2.8	0.2	-7
**CHEMBL190010**	-2.9	1.4	-0.0, 2.2, -0.4, -2.9, 3.4	-1.2	1.7	-7.9
**C10128921**	2.4	1.5	6.4, 4.2, 14.5, 13.7, 2.4	3.8	1.4	-7.5

Table S1. ABFE results for the BACE1 Tier 2 compound set. Compound: nomenclature as in Mysinger 2012 DUD-E paper; actives begin with `CHEMBL`. For each compound, both the more favorable and the less favorable overall binding free energies (BFE, kcal/mol) from the two independent runs are shown. Blocking uncertainties (kcal/mol) and pose-specific ABFE values (kcal/mol) are also presented for the more favorable result. Diff: difference between the two overall BFE values. Docking: docking score computed in this study.

	More Favorable BFE Run			Less Favorable BFE Run		
	**Overall BFE**	**Blocking Uncertainty**	**Pose BFEs**	**Overall BFE**	**Diff**	**Docking**
**CHEMBL74637**	-17.8	0.7	-15.6, -17.8, -10.4, -7.6, -7.6	-12.9	4.9	-10.3
**CHEMBL536029**	-17.4	1.4	-17.4, -14.4, -0.4, 11.2, -1.1	-12.5	4.9	-10.1
**CHEMBL332575**	-15.4	1.4	-15.4, -8.3, -2.1, -4.3, -5.9	-10.4	5	-10
**C01662799**	-14.3	1.2	-14.3, -7.8, -9.4, -8.8, -3.8	-11.5	2.8	-10
**CHEMBL180814**	-14.2	1.9	-14.2, -2.4, 4.1, 0.3, -5.4	-9.4	4.8	-9.9
**CHEMBL559513**	-13.9	1.7	-6.2, -13.9, 0.2, 5.4, -0.3	-11.2	2.7	-10.9
**CHEMBL181114**	-13.8	1.5	-9.3, -13.8, 10.0, 3.8, 3.9	-9.7	4.1	-9.9
**CHEMBL434081**	-13.1	1.3	-13.1, -11.7, -4.5, -3.8, -6.1	-9.9	3.2	-10.5
**CHEMBL1171949**	-12.8	2	-12.8, -7.4, -7.2, 2.0, -2.5	-8.3	4.5	-11.6
**CHEMBL150859**	-12	1.2	-11.9, -7.6, -4.9, 2.5, nan	-12	0	-10
**CHEMBL1094400**	-11.6	1	-11.6, -6.5, -4.4, -0.2, -6.0	-10.2	1.4	-11.1
**C08691668**	-11.4	0.9	-11.4, -2.9, -6.3, -3.9, -7.2	-7.3	4.1	-10
**CHEMBL311851**	-11	1.8	-11.0, -4.6, 2.1, 0.6, 6.1	-6.7	4.3	-10.8
**C09739152**	-10	1.7	-4.2, 0.8, 1.1, -10.0, -5.9	-7	3	-10
**CHEMBL75952**	-9.6	1.5	-7.5, -9.6, 5.0, 12.7, -2.3	-9.6	0	-10.5
**C39991461**	-9.6	1.7	-6.5, -2.8, -9.6, -5.4, -0.6	-5.8	3.8	-10
**C50039009**	-8.8	1.6	-8.7, -7.4, 0.4, 2.0, 9.1	-8.3	0.5	-9.9
**C04274093**	-8.8	1.2	-8.7, -6.3, -7.1, -0.1, 6.4	-5.3	3.5	-10.1
**CHEMBL104526**	-8.7	0.8	-6.0, -8.6, -6.9, -5.8, nan	-8.7	0	-10.2
**C66106409**	-8.6	1.6	-4.9, -1.1, -3.5, -8.6, -3.0	-6.7	1.9	-9.8
**C05151568**	-8.6	0.9	-5.1, -8.6, -3.5, -4.4, 3.2	-4.5	4.1	-10.6
**C60582423**	-8.6	1.1	-7.8, -3.6, 5.8, -8.4, 0.6	-8	0.6	-9.8
**C62038809**	-7.4	1.2	-0.6, -7.4, -4.2, -5.2, -2.8	-6.1	1.3	-10
**C57522130**	-7.3	0.9	2.5, -7.1, 2.1, -6.3, 2.4	-0.1	7.2	-10.1
**C04518765**	-7	1.2	-4.9, -0.6, -7.0, 4.4, 1.4	-6.7	0.3	-10.3
**C02508272**	-6.2	1.5	-6.2, 2.5, 5.0, 10.4, 7.9	-5.4	0.8	-10
**CHEMBL404747**	-6.1	1.9	-6.1, 4.8, 11.2, -0.5, 1.4	-5.9	0.2	-10.3
**C08994532**	-5.3	1.1	-5.3, 5.9, 11.4, 3.9, -0.1	-4.5	0.8	-9.9
**C39331065**	-5.1	1.6	-5.1, 1.9, 6.3, 7.6, 9.8	-1	4.1	-10
**C13451343**	-3.3	1.3	-3.3, 7.3, 7.0, 5.5, -0.4	0.1	3.4	-10.2

Table S2. ABFE results for the CDK2 Tier 1 compound set. See Table S1 for details.

	More Favorable BFE Run			Less Favorable BFE Run		
	**Overall BFE**	**Blocking Uncertainty**	**Pose BFEs**	**Overall BFE**	**Diff**	**Docking**
**CHEMBL147012**	-18.2	1.2	-9.7, -2.3, -6.3, -3.5, -18.2	-13.8	4.4	-8.7
**CHEMBL409892**	-15.7	0.7	-15.7, -7.6, -7.1, -6.5, -9.5	-11.6	4.1	-8.8
**CHEMBL436078**	-15.2	1.3	-15.2, -5.7, -6.4, -8.1, -3.4	-11.5	3.7	-9.1
**CHEMBL590109**	-15.2	1.5	-15.2, 1.2, -2.5, -5.2, 9.9	-10.6	4.6	-8.8
**C60767028**	-14.6	1.4	-11.0, -10.3, -14.6, -8.7, -3.4	-11.1	3.5	-9.3
**CHEMBL148977**	-12.8	1.1	-12.8, -4.4, -9.3, 3.7, -6.7	-9	3.8	-8.9
**CHEMBL434257**	-12.7	1.6	-12.7, -2.1, -0.7, -2.3, -6.0	-8	4.7	-8.6
**CHEMBL87472**	-12.4	1	-4.8, -3.3, -12.3, -6.0, -10.8	-12.3	0.1	-8.5
**CHEMBL254367**	-12	0.9	-10.4, -11.5, -11.6, 10.3, 8.2	-11.1	0.9	-9.1
**CHEMBL1081438**	-10.7	1.3	-10.5, -5.8, -2.0, -7.7, -9.8	-8.7	2	-9
**CHEMBL72077**	-10	2.5	-9.9, -0.8, -3.9, 9.3, 1.5	-6	4	-9.7
**CHEMBL360695**	-9.6	0.9	-7.8, 4.3, -3.0, -9.6, -2.5	-9	0.6	-8.1
**C35183061**	-9.3	1.1	-3.6, -9.3, -4.8, -2.7, -0.4	-5.1	4.2	-8.1
**CHEMBL524629**	-9.3	1.2	-8.4, -4.2, -1.8, -7.2, -9.2	-9.3	0	-8.4
**CHEMBL484990**	-9.2	1.4	-9.2, 5.5, -1.9, 3.5, 5.3	-6.7	2.5	-9.3
**CHEMBL1083785**	-9	1.6	-1.9, -3.3, -9.0, 2.4, 4.2	-3.7	5.3	-8.6
**C04861026**	-8.6	1.3	-8.6, -6.1, -1.3, -1.6, -2.2	-6.8	1.8	-8.5
**C20916853**	-8.3	1	-8.0, -7.5, -5.4, -6.3, -4.9	-7.7	0.6	-8.3
**C19212057**	-8.3	1.2	-8.3, -4.9, 0.3, -0.2, 2.7	-5.2	3.1	-8.4
**C06935213**	-8.2	0.7	-8.2, -6.8, -0.1, -6.3, -3.6	-7.4	0.8	-8.1
**CHEMBL48796**	-7.8	1.9	-7.8, 3.1, 0.1, -5.0, -2.5	-5.9	1.9	-9
**C44496935**	-7.7	1.5	0.6, -1.8, -2.9, -3.8, -7.7	-6	1.7	-8.2
**C17070426**	-6.7	1.5	-6.7, 1.7, -0.1, 0.3, 2.9	-4.7	2	-8.5
**C35781026**	-6.3	1.3	-6.3, -0.3, 1.5, 7.7, 3.7	-4.7	1.6	-9.5
**C05175710**	-6.3	1.9	0.7, -6.2, 2.5, 1.7, 1.1	-3.3	3	-8.1
**C44608137**	-6	0.8	-0.5, -5.0, -5.7, -3.8, -5.1	-5.5	0.5	-9.1
**C09468085**	-5.1	1.4	1.2, -5.1, 6.9, -2.3, 5.7	-3.3	1.8	-8
**C01251161**	-3	1.2	-0.1, 0.3, -1.1, 1.9, -2.9	-3	0	-9.1
**C66736964**	0.7	2	20.2, 11.4, 16.4, 13.6, nan	1.6	0.9	-8
**C42407232**	6.7	1.6	12.2, 24.7, 6.7, 10.6, 9.1	8.9	2.2	-8

Table S3. ABFE results for the CDK2 Tier 2 compound set. See Table S1 for details.

	More Favorable BFE Run			Less Favorable BFE Run		
	**Overall BFE**	**Blocking Uncertainty**	**Pose BFEs**	**Overall BFE**	**Diff**	**Docking**
**CHEMBL121694**	-17	1.8	-16.9, -6.3, 3.6, -0.9, -4.1	-15.4	1.6	-10.9
**CHEMBL200960**	-16.4	2.3	4.0, -1.9, -7.1, -3.1, -16.4	-9.7	6.7	-10.2
**C49054895**	-14.8	1.7	-14.8, -3.6, -0.1, 1.7, 0.7	-10.1	4.7	-10
**CHEMBL229036**	-13.8	2.5	-13.8, -7.4, -8.9, -6.8, 17.7	-11.3	2.5	-11.5
**CHEMBL228340**	-12.6	2.3	-12.6, -3.9, -11.1, -9.3, 3.1	-11.2	1.4	-11.4
**CHEMBL109999**	-12.4	1.6	-7.8, -6.8, -12.4, -1.7, -4.9	-7.9	4.5	-12
**CHEMBL19666**	-11.6	1.3	-11.6, -4.7, -7.0, -3.0, -0.7	-9.1	2.5	-10.7
**CHEMBL395970**	-10.5	1.3	-10.2, -9.1, -4.5, 6.5, -9.6	-9.8	0.7	-10.6
**CHEMBL277695**	-10.2	1.7	-8.0, -10.2, -4.8, -1.1, 2.2	-9.7	0.5	-12.1
**CHEMBL354456**	-9.8	2.7	-9.8, 0.7, 3.2, -3.3, -0.6	-4.1	5.7	-10.3
**C20776477**	-9.5	1.8	-1.2, 4.0, -0.8, 5.8, -9.5	-6	3.5	-10.1
**CHEMBL142546**	-9.3	1.1	-4.5, -9.0, -2.6, -8.5, -7.4	-9.1	0.2	-10.1
**C65170468**	-9.3	1.1	-9.3, -0.7, -3.0, 4.3, -2.8	-1.8	7.5	-10.4
**CHEMBL353213**	-9.3	2.2	-5.8, -2.3, -5.9, -6.9, -9.3	-4.3	5	-10.7
**CHEMBL207528**	-9.1	1	-5.4, 1.3, -3.3, -3.4, -9.1	-7.2	1.9	-11.2
**C06272150**	-9	2.3	-3.4, -7.0, -9.0, -1.0, -6.0	-4.4	4.6	-10.1
**CHEMBL167755**	-9	0.9	-9.0, -0.8, 0.3, -2.2, -4.4	-6.6	2.4	-9.9
**C20292354**	-7.6	1	-7.6, -1.7, -1.9, -1.9, 0.2	-7.5	0.1	-10.4
**C06444805**	-7.6	1.5	0.5, -5.8, -3.5, -6.4, -7.5	-4.5	3.1	-10.1
**CHEMBL121982**	-7.5	1.3	-5.8, -5.0, -3.1, 1.0, -7.4	-5.9	1.6	-10.5
**C64491035**	-7.3	0.9	-6.0, -3.5, -5.8, -5.9, -7.1	-7.2	0.1	-10.5
**CHEMBL101563**	-6.7	1.4	-6.6, -0.8, 2.1, -3.4, -5.0	-6.5	0.2	-10.5
**CHEMBL422387**	-6.6	1	-6.6, -2.2, -1.9, -1.2, -5.0	-4.4	2.2	-10.8
**C40148794**	-6.3	1.2	2.8, -5.4, -6.1, -3.3, 1.6	-3.1	3.2	-10.9
**C39958186**	-5.9	0.9	-3.6, -2.9, -0.2, -5.9, -0.2	-3.5	2.4	-10
**C36047434**	-5.9	1.4	4.3, 0.9, -0.3, -5.9, -4.4	-4.8	1.1	-10.2
**C66403456**	-5	1	-2.7, -1.8, 3.6, -3.2, -4.9	-4.6	0.4	-10
**C00845915**	-4.7	1.5	3.9, 1.0, -4.7, -0.3, -1.6	-2.6	2.1	-10.8
**C16951658**	-2.4	0.9	4.5, -0.7, 1.8, 2.1, -2.3	0.6	3	-10.1
**C38994577**	-2.1	0.8	1.1, -1.5, 4.8, -1.8, -0.2	-0.9	1.2	-11.1

Table S4. ABFE results for the thrombin Tier 1 compound set. See Table S1 for details.

	More Favorable BFE Run			Less Favorable BFE Run		
**Compound**	**Overall BFE**	**Blocking Uncertainty**	**Pose BFEs**	**Overall BFE**	**Diff**	**Docking**
**C38635562**	-15.9	1.1	-12.1, -9.1, -1.7, -6.9, -15.9	-12.3	3.6	-8.3
**CHEMBL254962**	-14.9	2.3	-14.9, -1.6, -7.8, -1.2, -1.0	-14.5	0.4	-8.0
**CHEMBL254759**	-12.6	1.5	-12.3, -6.3, -9.1, -2.9, -12.2	-10.9	1.7	-8.8
**C40780730**	-11.8	1.2	-11.8, 1.9, -3.8, -2.8, -4.4	-9.2	2.6	-8.8
**CHEMBL131999**	-11.3	1.0	-11.3, -2.0, -8.8, -2.9, -4.7	-6.9	4.4	-9.5
**CHEMBL182597**	-11.2	1.5	-0.2, 9.2, -3.6, -11.2, 0.2	-4.0	7.2	-8.4
**CHEMBL309670**	-10.9	1.0	-4.2, 9.3, -6.7, -0.2, -10.9	-8.6	2.3	-9.2
**CHEMBL373260**	-9.7	1.5	-9.7, -7.2, -5.7, -6.6, -4.2	-9.2	0.5	-9.3
**CHEMBL350955**	-9.0	1.7	-2.9, 0.1, -2.2, -9.0, 1.0	-5.9	3.1	-9.4
**CHEMBL184580**	-8.9	1.3	-8.9, -5.7, -4.3, 1.8, -2.5	-5.5	3.4	-8.6
**CHEMBL327189**	-8.5	1.2	-7.1, 1.3, -3.0, -8.5, -0.9	-6.2	2.3	-9.6
**C35636581**	-8.4	1.5	-8.4, 0.5, 5.9, -0.9, -4.7	-5.4	3	-8.2
**CHEMBL439678**	-8.2	1.7	0.8, -4.1, -8.2, -0.6, 0.9	-6.4	1.8	-8.1
**CHEMBL319443**	-7.8	1.5	-1.0, -7.8, -1.2, 0.7, -2.8	-7.5	0.3	-8.3
**C40879757**	-7.6	1.6	-7.6, -1.4, -0.9, -1.7, -3.1	-4.6	3	-8.1
**C39509237**	-7.5	1.3	-7.5, -1.0, 1.9, -0.7, -3.3	-2.7	4.8	-8.7
**C36532689**	-7.5	1.4	-3.0, 1.6, 0.4, -7.5, -0.6	-4.9	2.6	-8.7
**C12596203**	-7.5	0.8	-7.3, -5.4, -6.7, -4.6, -5.9	-7.4	0.1	-8.3
**C33097029**	-7.3	1.8	-5.9, -3.2, -4.1, -1.2, -7.3	-6.6	0.7	-8.1
**C49795141**	-7.2	1.2	-7.2, 0.1, -0.5, -3.9, -3.2	-4.6	2.6	-8.2
**CHEMBL191441**	-6.7	1.1	-6.5, -4.8, -6.0, -3.4, -3.1	-6.1	0.6	-9.2
**CHEMBL295863**	-6.3	2.5	0.9, 3.6, 0.1, -6.3, -0.0	-2.0	4.3	-8.2
**C14653295**	-5.7	2.4	3.4, -5.7, 1.6, -4.0, -1.1	-5.3	0.4	-8.2
**C04743775**	-5.2	1.6	-3.0, 0.1, -5.2, 3.2, -2.6	-3.2	2	-9.6
**C31118492**	-4.7	1.3	6.5, 0.1, -3.0, -4.7, -3.0	-3.1	1.6	-8.2
**C19345593**	-4.5	1.2	-2.7, 3.6, -4.0, 0.7, -4.2	-4.1	0.4	-8.6
**C66238808**	-4.5	0.9	-3.9, -2.8, -3.5, -1.5, -3.8	-4.0	0.5	-8.2
**CHEMBL382542**	-4.4	1.2	-2.7, -2.9, -0.2, -4.3, -2.5	-4.0	0.4	-8.8
**C40744515**	-4.1	1.1	-0.9, -2.0, -0.3, -1.6, -4.1	-0.6	3.5	-8.1
**CHEMBL112931**	0.2	2.1	8.5, 0.2, 4.7, 6.3, 7.1	6.0	5.8	-8.0

Table S5. ABFE results for the thrombin Tier 2 compound set. See Table S1 for details.
